# Adult-Onset Hypothyroidism Enhances Fear Memory and Upregulates Mineralocorticoid and Glucocorticoid Receptors in the Amygdala

**DOI:** 10.1371/journal.pone.0026582

**Published:** 2011-10-24

**Authors:** Ana Montero-Pedrazuela, Iván Fernández-Lamo, María Alieva, Inmaculada Pereda-Pérez, César Venero, Ana Guadaño-Ferraz

**Affiliations:** 1 Department of Nervous System and Endocrine Pathophysiology, Instituto de Investigaciones Biomédicas Alberto Sols, Consejo Superior de Investigaciones Científicas-Universidad Autónoma de Madrid, Madrid, Spain; 2 Department of Psychobiology, Universidad Nacional de Educación a Distancia, Madrid, Spain; Université Pierre et Marie Curie, France

## Abstract

Hypothyroidism is the most common hormonal disease in adults, which is frequently accompanied by learning and memory impairments and emotional disorders. However, the deleterious effects of thyroid hormones deficiency on emotional memory are poorly understood and often underestimated. To evaluate the consequences of hypothyroidism on emotional learning and memory, we have performed a classical Pavlovian fear conditioning paradigm in euthyroid and adult-thyroidectomized Wistar rats. In this experimental model, learning acquisition was not impaired, fear memory was enhanced, memory extinction was delayed and spontaneous recovery of fear memory was exacerbated in hypothyroid rats. The potentiation of emotional memory under hypothyroidism was associated with an increase of corticosterone release after fear conditioning and with higher expression of glucocorticoid and mineralocorticoid receptors in the lateral and basolateral nuclei of the amygdala, nuclei that are critically involved in the circuitry of fear memory. Our results demonstrate for the first time that adult-onset hypothyroidism potentiates fear memory and also increases vulnerability to develop emotional memories. Furthermore, our findings suggest that enhanced corticosterone signaling in the amygdala is involved in the pathophysiological mechanisms of fear memory potentiation. Therefore, we recommend evaluating whether inappropriate regulation of fear in patients with post-traumatic stress and other mental disorders is associated with abnormal levels of thyroid hormones, especially those patients refractory to treatment.

## Introduction

Morphological and functional brain adaptability is the basis for learning and memory. The integration of both external and internal signals as hormones modulates this plasticity. Thus, it has long been demonstrated that hormonal imbalances induce alterations in cognition, memory, and mood state [1]. A large number of epidemiological and experimental studies have shown that deficiencies in thyroid hormones (TH, triiodothyronine or T3 and thyroxine or T4) during critical periods of development induce irreversible brain damage affecting learning and memory capacities [2], [3]. Over the last few decades, a great effort has been made to implement programs for the prevention of neonatal hypothyroidism [4]. However, less attention has been paid to adult-onset hypothyroidism and its consequences, as it is a frequent condition in humans with the prevalence increasing with age [5], [6].

The main mechanism of TH action is modulation of gene expression in target cells through the binding of T3 to specific nuclear thyroid hormone receptors (TRs), mainly TRα1 and TRβ1, which act as aporeceptors and ligand modulated transcription factors [7]. Recent studies from various laboratories, including our own, using experimental animals demonstrate that adult-onset hypothyroidism impairs brain plasticity from molecular to macro-structural events with functional consequences. Adult hypothyroid rats show alterations in the expression of striatal synaptic plasticity proteins [8], reduced number of dendritic spines of cortical pyramidal neurons [9] and decreased adult hippocampal neurogenesis [10], [11], [12]. In addition, long-term potentiation (LTP) induction in hippocampal synaptic pathways [13], [14], [15] and some, but not all, hippocampal-dependent learning and memory processes are impaired in adult hypothyroid animals [12], [13], [14]. Interestingly, patients with hypothyroidism suffer not only from memory deficits [16], [17], [18], but also from emotional disturbances [5], [19]. Due to the common association between abnormal TH levels and psychiatric disturbances, it has been strongly recommended to evaluate thyroid function in patients with affective disorders [20].

Limbic regions involved in cognitive processes and emotional responses are sensitive to hormonal alterations. The amygdala (key to fear conditioning) shows high levels of TRs [21], [22] and of type 2 deiodinase, the enzyme responsible for the local conversion of T4 to nuclear active T3 [23], hence, the amygdala is likely to be an important target region for TH action. In spite of this, to our knowledge, no studies have analyzed the contribution of TH to Pavlovian fear conditioning, the most studied form of amygdala-related emotional memory [24], [25]. In this paradigm, an initially neutral conditional stimulus (CS, a tone or a context) is paired with an aversive unconditional stimulus (US, an electric foot shock). After single or repeated CS-US pairings, the CS alone elicits conditioned responses such as freezing, in that all movements cease, except those required to breathe [26]. The aversive stimulus induces the release of stress hormones, glucocorticoids (corticosterone in rats and cortisol in humans) that play an important modulatory influence on fear memory consolidation acting through glucocorticoid receptors at the lateral/basolateral (LA/BLA) nuclei of the amygdala (for review see [27]). In addition, the conditioned response can be reduced or extinguished by the repeated presentation of the CS in the absence of the US. Therefore, animal studies using Pavlovian fear conditioning and extinction can help to investigate the pathogenesis of human anxiety disorders [28], such as post-traumatic stress disorder (PTSD) [28], [29], [30], given that this disorder involves learned fear [31].

In the present study we have used the Pavlovian fear conditioning paradigm in order to explore the effect of adult-onset hypothyroidism on emotionally driven learning and memory. Our results show that euthyroidism is necessary for proper processing of emotional memories, as hypothyroidism enhances fear memory, delays extinction and exacerbates spontaneous recovery of fear memory. Our studies also suggest an abnormal glucocorticoid signaling in the amygdala as an important pathophysiological mechanism in hypothyroidism. The relevance of our findings is underlined by the clinical significance of TH in affective disorders and their potential role in PTSD and their treatment.

## Results

### Adult-Onset Hypothyroidism Potentiates Contextual and Auditory Fear Memory

**Figure 1:**
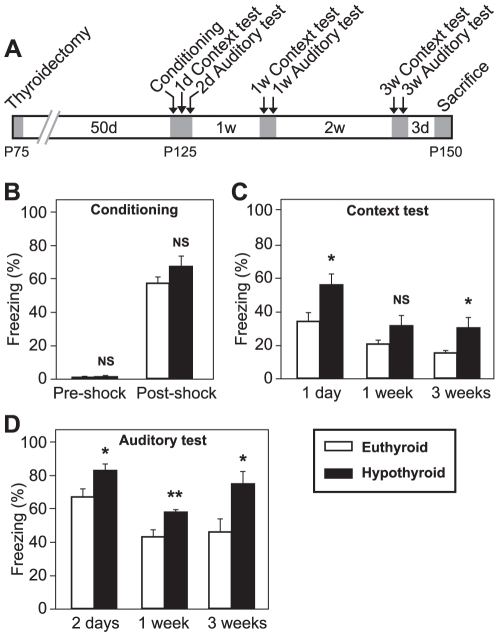
Contextual and auditory cued fear conditioning tests in euthyroid and adult-onset hypothyroid rats. (**A**) Schematic representation of the experimental design. Shadowed areas indicate periods of animal manipulations. (**B**) Conditioning session for auditory and contextual cues. No differences were found in neither pre-shock nor post-shock periods. (**C**) Contextual fear test sessions 1 day, 1 week and 3 weeks after conditioning. H rats showed higher freezing levels than E rats, which reach statistical differences 1 day and 3 weeks after conditioning. (**D**) Auditory cued fear testing 2 days, 1 week and 3 weeks after the conditioning session. H rats showed higher freezing levels during the tone period than their E counterparts at every test session after conditioning. Plot bars show means ± SEM. *, p<0.05; **, p<0.01; NS, no significant. n = 8 per group.

To evaluate the effects of adult-onset hypothyroidism on functional alterations on fear memory, we used Pavlovian contextual and auditory delay fear conditioning tests following the experimental design explained in Methods (Experiment 1) and Figure 1A. Auditory delay fear conditioning depends on the integrity of the amygdala, whilst contextual fear conditioning is sensitive to both amygdala and hippocampal lesions [32]. In the conditioning session euthyroid (E) and hypothyroid (H) rats showed similar freezing levels during pre and post-shock intervals (t_14_ = 0.178, *p* = 0.862 and t_14_ = 1.576, *p* = 0.137, respectively; Figure 1B). These results indicate that hypothyroidism does not interfere with conditioning acquisition.

For contextual fear conditioning testing, the rats were placed inside the original conditioning chamber, but no foot shock was delivered. Rats' behavior was registered 1 day, 1 week and 3 weeks after conditioning. Repeated-measures analysis showed a significant effect for session and group (*p*<0.001 in both cases). H animals displayed higher freezing levels in contextual fear memory as compared to E rats (a 64% increase 1 day, a 53% increase 1 week and a 95% increase 3 weeks after conditioning). Statistically significant differences between groups were found in sessions performed 1 day and 3 weeks after conditioning (t_14_ = 2.544, *p* = 0.023 at 1 day; t_14_ = 1.576, *p* = 0.137 at 1 week and t_14_ = 2.260, *p* = 0.040 at 3 weeks sessions; Figure 1C).

To test the auditory cued fear memory the rats were located in a new context changing the environment where conditioning and contextual test sessions were performed. Rat's behavior was registered for pre-tone and tone periods 1 day after each contextual fear conditioning test. Freezing levels in the auditory testing boxes before tone reappearance remained low in both groups, indicating that the new context differs significantly to the original context and was not recognized by the animals. There were no differences between groups during pre-tone period in any case (*p*>0.15, not shown). During tone period, repeated-measures analysis showed a significant effect for session and group (*p*<0.001 in both cases). Repeated-measures analysis showed a significant effect for session and group (*p*<0.001 in both cases). H animals showed a statistically significant increase in freezing levels at every test session as compared to their E counterparts (24% increase, t_14_ = 2.421, *p* = 0.030 at 2 days; 35% increase, t_14_ = 3.064, *p* = 0.013 at 1 week and 61% increase, t_14_ = 2.602, *p* = 0.020 at 3 weeks sessions; Figure 1D). These results indicate that adult-onset hypothyroidism leads to long lasting robust fear memory.

### Adult-Onset Hypothyroidism Delays Extinction and Exacerbates Spontaneous Recovery of Auditory Cued Fear Memory

**Figure 2:**
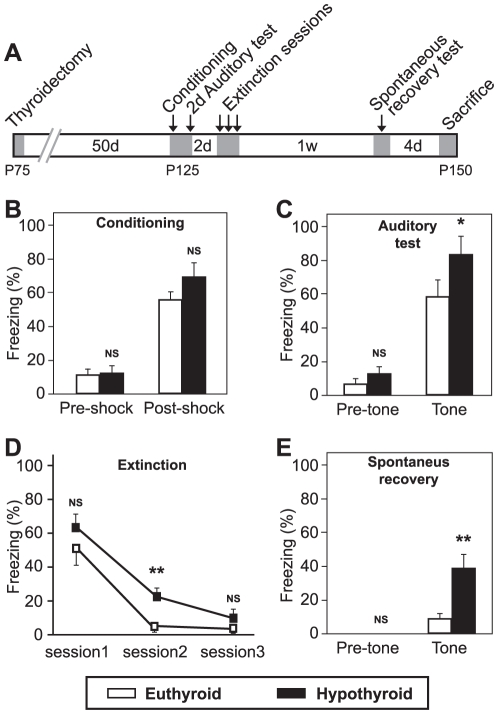
Auditory cued fear conditioning training, memory, extinction and spontaneous recovery tests in euthyroid and adult-onset hypothyroid rats. (**A**) Scheme showing the experimental design for Experiment 2. Shadowed areas indicate periods of animal manipulations. (**B**) Conditioning session. No differences were found in neither pre-shock nor post-shock periods. (**C**) Auditory cued fear test session 2 days after conditioning. H animals showed a significant increase in conditioned behavior. (**D**) Extinction sessions for the auditory cue fear memory. The extinction of fear memory was delayed in H animals. (**E**) Re-exposition to the tone for the assessing of spontaneous recovery of fear memory. H rats showed a remarkable increase (four-fold) in fear response as compared to E rats. Bars show mean ± SEM. *, p<0.05; **, p<0.01; NS, no significant. n = 7 for E rats and n = 8 for H rats.

In Experiment 1 we observed that hypothyroid rats displayed increased auditory fear memories even 3 weeks after conditioning, at a time when controls show a clear decrease in freezing levels that may be due to forgetting or extinction processes, or both. Since procedures in experiment 1 were not designed to evaluate extinction, we decided to explore whether the long lasting effects of hypothyroidism on auditory cued fear memory were due to failure in extinction learning to the repeated presentation of the conditioning tone in the absence of shock (Experiment 2, Figure 2A).

First, the animals were again conditioned to the tone and tested 2 days after conditioning. The results further confirm that adult-onset hypothyroidism does not interfere with basal fear levels and conditioning acquisition (pre-shock: t_13_ = 0.198, *p* = 0.846; post-shock: t_13_ = 1.319, *p* = 0.210; Figure 2B), but induces a potentiation of auditory fear memory (43% increase, t_13_ = 2.343, *p* = 0.036; Figure 2C).

We then analyzed the effects of hypothyroidism on fear extinction. Both groups of animals showed a decrease in freezing behavior across the 3 extinction sessions. However, memory extinction was more rapid in E animals that showed almost a complete extinction of fear (freezing level <20%) at session 2, while this occurred in H animals at session 3 (Figure 2D). Repeated-measures analysis showed significant effects for session (*p*<0.001) and group (*p* = 0.002). Differences between groups were restricted to the second extinction session (t_13_ = 3.287, *p* = 0.007). These results indicate a delay in fear extinction in H rats.

Extinguished fear conditioned responses often reappear after a time without further extinction training through spontaneous recovery. One week after the last extinction session, the animals were re-exposed to the auditory cue for measurement of the spontaneous recovery of emotional memory. H animals exhibited a greater than four-fold increase in freezing behavior as compared to E rats (t_13_ = 3.332, *p* = 0.005).

### Adult-Onset Hypothyroidism Alters Basal and Fear Conditioned-Induced Plasma Corticosterone Levels without Affecting the Weight of Adrenal Glands

**Figure 3:**
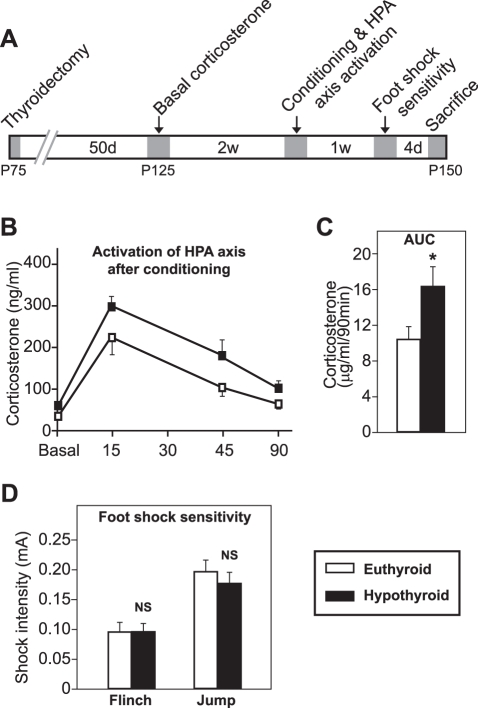
Plasma corticosterone levels and sensitivity to foot shocks in euthyroid and hypothyroid rats. (**A**) Scheme showing the experimental design for Experiment 3. Shadowed areas indicate periods of animal manipulations. (**B**) Dynamics of the plasma corticosterone response to training in the cued fear conditioning task (0, 15, 45 and 90 min after training). (**C**) Area under the curve (AUC) analysis showed significantly increased corticosterone levels in H rats as compared to E animals. (**D**) Sensitivity to foot shocks was not altered in H animals. No differences were found in either flinching or jumping behaviors. Bars show mean ± SEM. *, p<0.05; NS, no significant. n = 7 per group.

We decided to analyze if fear memory potentiation in H rats could be related to alterations in post-conditioning hypothalamus-pituitary-adrenal (HPA) axis reactivity. First, basal plasma corticosterone levels were measured in naïve animals. H animals showed an increase in basal corticosterone levels as compared to euthyroid animals (E = 35.7±4.6 ng/ml; H = 60.7±5.3 ng/ml; t_12_ = 3.545, *p*<0.004). To analyze post-conditioning HPA axis reactivity, the corticosterone response curve was studied at different time points after foot shock delivery, which acts as a stressor (Figure 3B). Repeated-measures analysis revealed significant effects of sampling time (*p*<0.001) and group (*p* = 0.003). Area under the curve (AUC) analysis showed an increase in corticosterone response in H animals as compared to E rats (t_12_ = 2.268, *p* = 0.043; Figure 3C). This result shows that corticosterone stress response after fear conditioning is enhanced in adult-onset hypothyroidism. No significant changes in relative weight of adrenal glands were observed between both groups of animals (Experiment 3, t_12_ = 1.018, *p* = 0.349; Table 1).

**Table 1:** Thyroid Hormones Levels, Body Weight (BW) Gain and Adrenal Glands Relative Weight in Euthyroid and Hypothyroid Animals.

	Plasma T3 (ng/ml)	Plasma T4 (ng/ml)	BW gain (g)	Adrenal glands relative weight (g/100 g BW)
Euthyroid rats	0.61±0.05	25.78±1.78	126.8±5.4	0.126±0.012
Hypothyroid rats	0.16±0.02***	1.50±0.17***	26.8±4.7***	0.143±0.011^NS^

The relative adrenal glands weight refers to absolute adrenal glands weight/100 g BW. Data represent mean values ± SEM. Significant differences as compared to the euthyroid group are shown as *** p<0.001. NS, no significant. For TH levels and BW gain, n = 30 for E rats and n = 31 for H rats. For adrenal glands relative weight, n = 7 for each E and H groups.

### Adult-Onset Hypothyroidism Does Not Alter Pain Sensitivity

The results of the foot shock sensitivity test demonstrated that thyroidal status did not modify the reactivity to the shock, which was determined by measuring pain thresholds for two typical foot shock-induced behaviors: flinch (t_12_ = 0.891, *p* = 0.390) and jump (t_12_ = 0.172, *p* = 0.261; Figure 3D).

### Adult-Onset Hypothyroidism Does Not Modify LA/BLA Volume

**Figure 4:**
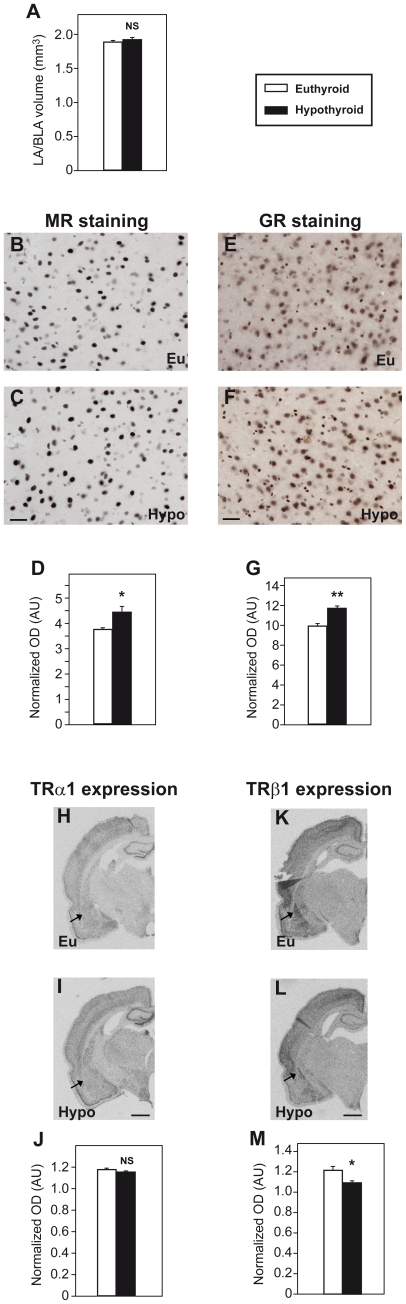
Expression of glucocorticoid, mineralocorticoid and thyroid hormone receptors in the lateral and basolateral nuclei of the amygdala in euthyroid and hypothyroid rats. (**A**) Amygdala volume estimation in E and H rats. No differences were found between groups. Representative photomicrographs (40×) of MR (**B, C**) and GR (**E, F**) immunohistochemistry in LA/BLA in E (Eu) and H (Hypo) animals. Scale bar, 20 µm. Histograms show the mean gray values of GR (**D**) and MR (**G**) obtained for E and H animals (n = 4 per group). GR and MR staining were increased in H rats. Panoramic views of isotopic *in situ* hybridization for TRα1 (**H, I**) and TRβ1 (**K, L**) in coronal sections of the brain of E (Eu) and H (Hypo) animals. Scale bar, 0.15 cm. Arrows point to the amygdala. Histograms show the mean gray values in arbitrary units (AU) obtained for E and H rats for TRα1 (**J**) and TRβ1 (**M**) mRNA expression (n = 3 per group). No differences were found between groups for TRα1. TRβ1 expression was reduced in H rats. Plot bars show means ± SEM. *, p<0.05; **, p<0.01; NS, no significant.

The exaggerated emotional memory displayed by H animals could be related to morphological and/or biochemical changes in the amygdala [24]. Variations in amygdala volume have been associated with alterations in fear memory processing in experimental animals [33] and with several neurological and neurodegenerative disorders in humans [34]. However, in this particular instance, no differences were found in the mean volume of the LA/BLA between E (mean ± SD = 1.89±0.22 mm^3^) and H (1.93±0.23 mm^3^) rats (t_6_ = 0.247; *p* = 0.813; Figure 4A).

### MR and GR Expression in the LA/BLA is Increased by Adult-Onset Hypothyroidism

Given that H rats showed an enhanced corticosterone stress response after fear conditioning, we decided to gain insight into the possible mechanisms involved in the pathophysiology of hypothyroidism by analyzing the expression of mineralocorticoid and glucocorticoid receptors (MR and GR) in LA/BLA, an important region for glucocorticoids signaling involved in auditory fear memory [35]. The expression of MR and GR was analyzed in adult E and H naïve rats at 120 days of postnatal age (P120) using immunohistochemistry. The specificity of the antibodies used was shown previously by others [36], [37]. The overall aspect and distribution of GR-immunoreactive (-ir) and MR-ir cells fitted well with earlier descriptions [38]. Moderate signal was found in the LA/BLA for both MR and GR and both markers showed a nuclear staining, with a wide range of signal intensity, from weak to very strong. The nuclear staining showed a speckled pattern with a clear nucleolus (Figure 4 B–C and E–F). The densitometric analysis of overall staining for expression of both MR and GR in LA/BLA showed an increase in H animals as compared to E rats (t_6_ = 4.904, *p* = 0.003; Figure 4D and t_6_ = 2.880; *p* = 0.028; Figure 4G, respectively), suggesting that MR and GR mRNA expression are upregulated in adult-onset hypothyroidism.

### TRβ1 mRNA Expression, but no TRα1, is Decreased by Adult-Onset Hypothyroidism in the LA/BLA

We also investigated the influence of hypothyroidism on the expression of TRβ1 and TRα1 (the most abundant TRs in the mature brain), as unliganded TRs repress the transcription of positively modulated T3 target genes. TRα1 and TRβ1 mRNA expression was analyzed by *in situ* hybridization in the forebrain of E and H rats. Our results showed differential expression patterns for each isoform (data not shown) as previously described [21], [22]. No differences were found in the general pattern of expression for neither TRα1 nor TRβ1 between E and H animals. TRα1 mRNA highest expression levels were found in the dentate gyrus of the hippocampal formation and layer II of pyriform cortex. Overall, TRβ1 mRNA showed lower levels of expression than TRα1 with highest expression levels in layers II, III and IV of the cingulate and insular cortex, perirhinal cortex, lateral septal nucleus and amygdala.

In the amygdala, TRα1 mRNA showed a uniform distribution throughout the different nuclei (Figure 4 H–I). TRβ1 mRNA was expressed in all amygdaloid nuclei except in the central amygdaloid nucleus (Figure 4 K–L). In the LA/BLA TRα1 expression showed no differences between groups (t_4_ = 1.955, *p* = 0.122; Figure 4J), while TRβ1 expression was significantly reduced in H animals as compared to E rats (t_4_ = 3.036, *p* = 0.032, Figure 4M).

## Discussion

Our findings provide the first data on the influence of adult-onset hypothyroidism on emotional learning and memory. They show that hypothyroidism not only potentiates fear memory, but also delays extinction and facilitates the return of the extinguished behavior as observed in the spontaneous recovery test. Hypothyroidism also enhances the stress response to foot shock delivery, while in the LA/BLA increases in MR and GR expression and decreases in TRβ1 expression levels are observed. These results suggest that TH play a key role in the mechanisms of fear memory processing.

We evaluated acquisition of fear conditioning and fear memory by analyzing the response to different CS, context and tone, in the same animals (Experiment 1). Hypothyroidism does not affect the learning processes necessary for forming an aversive association between contextual or auditory information and the aversive stimulus. However, hypothyroidism potentiates the memory of this association over time, inducing a long-lasting fear memory. Given that after thyroidectomy a long period of time (at least 2 weeks) is required to reach hypothyroid TH concentrations in the brain [39], the experimental design to assess whether the absence of TH affects consolidation and/or retrieval of fear memory is problematic. In any case, the fact that hypothyroidism potentiates fear memory points to alterations in amygdala functioning, although a hippocampus-dependent effect cannot be excluded. Hypothyroid rats showed low freezing levels in the context prior to tone testing, suggesting that the increased freezing observed during the tone tests was associatively mediated and not due to generalized fear. The possibility that the enhanced fear memory observed in H animals could be related to alterations in sensory abilities was discarded, as hypothyroidism did not modify the pain sensitivity to foot shock intensity. In addition, previous studies from our group using the same experimental model of adult-onset hypothyroidism did not show alterations in auditory system functionality evaluated with auditory brainstem response test [13]. Hence, we evaluated extinction and spontaneous recovery of auditory fear memory (Experiment 2). Although H rats were able to extinguish the aversive memory throughout the successive extinction trials, they showed a slower extinction curve. Importantly, the reemergence of conditioned responses after extinction training occurred at very low levels in E rats, while H rats showed a remarkable exacerbation of spontaneous recovery of the aversive memory. Interestingly, deficient extinction learning has also been found in patients with PTSD [40], [41], [42] and fear extinction is considered an important mechanism in PTSD exposure therapy in humans [31]. Given that extinction is considered a new learning in which the prefrontal cortex (PFC) inhibits the expression of the original fear memory [43], [44], it may be hypothesized that the delayed extinction observed in H rats is due to a deficit of inhibitory control of PFC on amygdala activation. Alternatively, the observed delay in fear extinction may be affected by the strength of prior auditory cued fear conditioning [45].

The pathological mechanisms of hypothyroidism underlying potentiation of fear memory could be related to an enhancement of amygdala activity due to changes in gene expression. T3 modulates gene expression by the binding to specific TRs. In hypothyroidism, altered transcription of T3 target genes is mainly due to transcriptional repression by unliganded TRs [46]. Previous studies have demonstrated that hypothyroidism induces region-specific changes in TRs expression [47], [48]. However, to our knowledge, no studies have examined the effect of hypothyroidism on TRs expression in the amygdala, a brain region that shows prominent levels of TRs [22]. We analyzed the effects of hypothyroidism on the expression of TRα1 and TRβ1 in LA/BLA nuclei, which are critically involved in fear memory [24], [25], [49]. The quantitative expression analyses showed a specific reduction of TRβ1 expression in LA/BLA in H animals. As hypothyroidism would lead to an increase of unliganded TRβ1, it is tempting to speculate that a reduction in TRβ1 expression in H rats may decrease gene repression as an attempt to compensate T3 deficiency.

The enhanced fear displayed by H rats could also be ascribed to a generalized action of TH deficiency in the brain. Nevertheless, this possibility is unlikely as different circuits are involved in different learning and memory tasks and adult-onset hypothyroidism does not interfere with all types of learning and memory processes, as indicated by our previous and other experimental and clinical studies [12], [13], [14], [17], [18]. Based on these and present results, it is tempting to speculate that under TH deficiency there is a hierarchy in preserving memory capacities related to survival, as fear memories, in order to avoid the situations that induced them, although this preservation leads to a potentiation of fear memory.

Diverse evidence indicates that glucocorticoid modulation of memory consolidation, retrieval and extinction of emotionally arousing experiences involves a selective activation of corticosteroid receptors (GR and MR) in the LA/BLA [25], [50]. Glucocorticoids are released after HPA axis activation in response to aversive stimuli [27]. Herein, H rats showed higher corticosterone levels after foot shock delivery that could account for their enhanced fear memory. Previous studies found that glucocorticoid release after fear conditioning training plays a key role in the consolidation of the aversive memory [51], [52]. In addition, subcutaneous injection of corticosterone or dexamethasone just after training increases the freezing response in auditory fear conditioning tests [53], [54]. In the present study, the levels of GR and MR were increased in the LA/BLA of H animals. Hence, the novel finding of higher corticosterone plasma levels after fear conditioning together with an increased amygdalar GR levels in H rats may explain their altered emotional processing. Although we did not discriminate whether more cells express MR and GR or the expression levels per cell are increased, these emotional alterations are probably due to an increase in the number of liganded corticosteroids receptors.

Effects of glucocorticoids on the hypothalamus-pituitary-thyroid (HPT) axis and of TH on the HPA axis are reciprocal and inversely related [55], [56]. However, mechanisms underlying these interactions are poorly understood and indirect, affecting hypothalamic and pituitary modulation as well as hormonal metabolism. In addition, compensating mechanisms may exist, however, reported results have been conflicting. In hypothyroid experimental animals, no changes or decreased basal plasma corticosterone levels have been reported. This variability is possibly due to the differences in the experimental methods used as the techniques to induce hypothyroidism (i.e. propylthiouracil may alter adrenal steroidogenesis and attenuate ACTH-induced corticosterone response) or to the use of anaesthesia [57], [58], [59], [60]. It is important to indicate that the selective surgical thyroidectomy used here resulted in no significant changes in the relative weight of adrenal glands and in a mild increase of basal corticosterone levels, far from stress-induced levels. In humans, basal serum cortisol levels are mostly in normal ranges in primary and secondary hypothyroidism, as well as in hyperthyroidism, due to compensated cortisol secretion and metabolism owing to the intact HPA feedback relationship [55], [56]. However, interestingly, increased basal cortisol levels have been found in patients with primary hypothyroidism [61] and in thyroidectomyzed patients with differentiated thyroid cancer during acute hypothyroidism induced by T4 withdrawal [62], as occurs in the thyroidectomized rats from this study. The experimental model used in this study could be considered as a model to analyze the consequences of the clinical protocols that include hypothyroidism for several weeks in the treatment and follow-up of differentiated thyroid cancer as patient preparation for ^131^I-scanning after thyroidectomy.

The effects of TH deficiency on stress-induced glucocorticoids release are poorly understood. In our study, stress-induced release of corticosterone is clearly upregulated in H animals. Several points in the corticosterone production and metabolism could be altered involving acute stress responses. Interestingly, physical stress (foot shock) causes a decrease in TH in adult rats [63].

Basal cortisol levels in hypo- and hyperthyroid patients are not considered clinically relevant, due to the minor changes observed. Our present results suggest that under stressful situations the hypothyroidism-induced dysregulation of the HPA axis is greatly intensified and should be taken into account. Further studies should be performed to better understand the mechanisms underlying the interaction between the HPA-HPT axes under fear conditioning learning in normal and pathological situations.

Our results suggest an increased susceptibility to traumatic and emotional memories induced by adult-onset hypothyroidism. Fear memory potentiation in H rats would involve maladaptation, as emotional information of moderate intensity would trigger an exaggerated behavioral response. Inappropriate regulation of fear can lead to negative physical and psychological outcomes such as phobia, panic disorder or PTSD. Many treatments for these disorders are based on progressive extinction of fear memories through repeated exposure to the conditioned stimulus [31]. Thus, hypothyroidism-induced inability to properly extinguish fear memories and the strong recall of these memories could increase the risk for traumatic illnesses and resistance to extinction treatments. Interestingly, in PTSD patients, both increased and decreased TH levels have been found [64], [65], [66], [67]. Moreover, it has been suggested that the type of traumatic experience may influence the response of the thyroid system [67]. To our knowledge, only one study in the literature investigated the effects of hypothyroidism on PTSD in humans [66]. In this study, Kamoi et al. observed that a number of symptoms related to PTSD two months after an earthquake were higher in patients with Hashimoto's disease (a common cause of primary hypothyroidism). In relation to this, it will be very interesting to determine in future studies if the alterations characterized in this study in H rats could lead to permanent or transient damage, as we have observed in previous studies [12], [13].

Investigating factors, like hypothyroidism, that influence the acquisition and maintenance of Pavlovian fear conditioning in experimental animals will help to elucidate the psychobiological mechanisms that modulate the development and severity of some PTSD symptoms and their treatment.

## Materials and Methods

### Ethics Statement

All experimental procedures were performed following the European Union Council guidelines (86/609/EEC and 2003/65/EC) and Spanish regulations (BOE 252/2005, 34367-91) for the use of laboratory animals in chronic experiments, and were approved by the ethics committee of our institution (CSIC; approval numbers PN2007-116 and PN2010-55). All surgery was performed under anesthesia (intraperitoneal injection of a mixture of ketamine 100 mg/kg and medetomidine 0.1 mg/kg), and all efforts were made to minimize suffering.

### Experimental Animals

Adult male Wistar rats were housed in temperature controlled animal quarters with automatic light/dark cycles of 12/12 h. Thyroidectomized hypothyroid (H) rats and sham-operated euthyroid (E) rats were analyzed. Thyroidectomy was performed at P75 following a well established protocol in our laboratory, which leaves the parathyroids intact and where H rats are fed a low iodine diet starting the day of surgery [12], [13], [68]. To confirm thyroidal status, all rats were weighed twice a week and plasma T3 and T4 levels were measured from every animal after sacrifice (P150, except animals for histological studies that were sacrificed at P125) as previously described [69]. No differences were found in T3 or T4 levels between both ages (n = 30 for E rats and n = 31 for H rats). Thyroidectomized rats had significantly lower circulating T3 and T4 levels and a significantly reduced body weight (BW) gain since thyroidectomy than that of E animals (Table 1). This indicates that our experimental animals are adequate to study the effects of adult-onset hypothyroidism on emotional memory. Adrenal glands were excised and weighted (Table 1; n = 7 per group). The relative adrenal glands weight refers to absolute adrenal glands weight/100 g BW.

### Delay Fear Conditioning

The animals were housed in groups of 2–3 animals/cage. They were handled for 2 min daily for 3 days before each experiment for habituation to experimental manipulations. Tests were always conducted between 08.00 and 13.30 h to avoid the influence of circadian hormonal fluctuations. All tests began at P125, 50 days after surgery. Conditioning and testing took place in a rodent observation cage using a shock generator (model LI100–26 Shocker, LETICA I.C., Madrid, Spain) as previously described [70], [71]. The observation cage (30×37×25 cm) was placed in a sound-attenuating chamber. The side walls of the observation cage were constructed of stainless steel and the back walls and doors were constructed of clear Plexiglass. The floor consisted of 20 steel rods through which a scrambled shock from could be delivered. The observation cage was cleaned with a 0.1% acetic acid solution before and after each session. Ventilation fans provided a background noise of 68 dB and a 20 W white light bulb illuminated the chamber.

Firstly, we explored in Experiment 1 (Figure 1A) possible functional alterations in hippocampal and/or amygdala-dependent fear memories in H animals on contextual and auditory delay fear conditioning using a foot shock as US (n = 8 for each E and H groups). On the conditioning day each rat was transported from the colony room to the laboratory (situated in adjacent rooms) and placed in the conditioning chamber. After 3 min, three tone-shock pairings were delivered with an inter-shock interval of 60 s. The tone (85 dB sound at 1000 Hz) was presented for 20 s and at the end of each tone an electric foot shock was delivered (1 s, 0.4 mA, constant current). The rats were removed from the conditioning chambers 30 s after the final shock presentation, and returned to their home cages. Thus, a conditioning session lasted ∼5.5 min. Testing for contextual fear conditioning was performed 1 day, 7 days (1 week) and 21 days (3 weeks) after conditioning. At testing, rats were placed back in the same chamber as used for conditioning but in the absence of shock or tone, for an 8 min context test. Testing for auditory fear conditioning was performed 2 days, 8 days (1 week) and 22 days (3 weeks) after conditioning. Rats were placed in the absence of shock in a novel context (same cages but with different walls, floor and background odor) in the absence of the conditioning tone (3 min; pre-tone period) and then re-exposed to the tone (5 min; tone period). Using a time-sampling procedure the behavior was evaluated in each experimental session and each rat was scored blindly as either freezing or active every 2 s. Freezing was defined as behavioral immobility except for movement required for breathing. This freezing response is considered as a fear index. At conditioning, behavioral scores were noted for the 3 min period prior to shock (pre-shock period) and for the 2.5 min period starting immediately after presentation of the first shock (post-shock period). Scores for each of these periods were analyzed separately. At testing for auditory fear conditioning the scores for the pre-tone and tone periods were also considered separately. At testing for contextual fear conditioning the scores for the total 8 min context test were analyzed. To perform the statistical analysis the data were transformed to a percent of total time.

Secondly, we evaluated in Experiment 2 (Figure 2A) the effects of adult-onset hypothyroidism on the extinction and spontaneous recovery of auditory delay fear memory in an independent group of rats (n = 7 and n = 8 for E and H groups, respectively). The conditioning and auditory test session (2 days test session) were conducted and analyzed as explained in Experiment 1. Three extinction sessions were performed on consecutive days starting 2 days after the test session. In the extinction sessions, rats were placed in the absence of shock in a novel context during 10 min. Each minute consisted in 40 s in the absence of the conditioning tone and 20 s of the re-exposure to the tone (same tone as in training). During extinction no shock was delivered. One week after the last extinction session, an auditory test session was performed to evaluate spontaneous recovery of the fear memory.

### Plasma Corticosterone Measurements

Plasma corticosterone levels were measured in E and H animals (Experiment 3, Figure 3A; n = 7 per group). Blood samples (∼300 µl) were obtained by tail-nick procedure whilst the animal was smoothly wrapped with a cloth and the blood introduced into ice-cold EDTA capillary-system tubes (Sarstedt, Germany). To obtain basal levels, a sample was taken between 09.00h and 09.30h, two weeks before training the animals in the fear conditioning. To quantify corticosterone levels induced by fear conditioning procedure blood samples were taken at different time-points after auditory fear conditioning training (15, 45 and 90 min). Subsequently, blood samples were centrifuged (3000 rpm for 20 min, at 4°C) and plasma was stored at −70°C. Corticosterone was measured using a radioimmunoassay kit (Coat-A-Count, Diagnostics Products Corporation; CA, USA; sensibility 5.9 ng/ml; intra-assay variability 3.2–4.5%).

### Foot Shock Sensitivity Test

To assess if hypothyroidism modified the sensitivity to foot shocks in our experimental conditions (Experiment 3, Figure 3A; n = 7 per group), each rat was placed individually in a conditioning chamber different to that used for conditioning. After 120 s each rat received an ascending series of 1 s foot shocks, separated by 20 s, in 0.05 mA increments from 0 mA until the animal showed the first signs of discomfort (moving backwards and flicking their hind legs, scored as flinch) and pain (defined as the animal's paws leaving from the grid floor, jumping and vocalization, scored as jump). The shock intensity that elicited each of the two reactions was assessed. A lower shock intensity inducing the response is interpreted as greater sensitivity to the foot shock.

### Histological Analyses

#### Amygdala Volumetric Analysis

The anatomy of the amygdala was analyzed by Nissl staining in parallel series to that used for immunohistochemical analyses (see below) using the atlas of Paxinos and Watson [72]. The Cavalieri method on point counts was used for the stereological estimation of the LA/BLA volume. An average of 180–240 points was counted per LA/BLA in a total of eight sections per animal. The mean CEs were 2.4% for both groups.

#### Immunohistochemistry of Corticosteroid Receptors

The influence of thyroidal status on nuclear receptors expression was analyzed in groups of P125 naïve rats (n = 4 for each H and E groups) in order to avoid the stress from behavioral studies. Immunohistochemistry was performed as previously described [36], [73]. Briefly, rats were sacrificed at P125 (50 days after surgery) and transcardially perfused with 4% paraformaldehyde in 0.1 M PB. The brains were serially sectioned on a vibratome at 50 µm in the coronal plane. GR and MR expression was analyzed in the LA/BLA at −1.50 to −3.60 from Bregma on free-floating sections using specific antibodies (rabbit antibody anti-GR, 1∶300; sc-8992, Santa Cruz Biotechnology Inc., Santa Cruz, CA, USA [37]; mouse anti-MR, 1∶300; rMR1-18 1D5, developed by Celso Gomez-Sanchez [36], obtained from the Development Studies Hybridoma Bank developed under the auspices of the NICHD and maintained by the University of Iowa, Deparment of Biolological Sciences, Iowa City, 1A 52242). Biotinylated secondary antibodies (Vector Laboratories, Burlingame, CA, USA) were used at a 1∶200 dilution. The immune signal was developed using the Vectastain Elite ABC Kit and DAB Peroxidase Substrate kit (Vector Laboratories), with nickel intensification.

Nuclear receptors relative expression was studied under brightfield illumination with an Olympus BX51 microscope using planapochromatic lenses. To estimate the relative GR-ir and MR-ir signal in both experimental conditions, optical densitometric analysis of an unbiased systematic uniform random sampling was used [74]. Briefly, 60–70 microphotographs were taken in the LA/BLA from six sections of each animal with an upper guard distance of 7 µm using an Olympus DP70 digital camera with an Olympus UPlanSApo 100X oil-immersion lens (1.40 numerical aperture). Stereological measurements were carried out in this microscope with the help of an interactive computer system comprising a high-precision motorized microscope stage, a 0.5-µm resolution microcator (Heidenhain VZR401) and the CAST stereological software package (Visiopharm, Hørsholm, Denmark, and Olympus España). Images were collected in TIFF format and transformed to 8-bit gray and using the image analysis software ImageJ (Beta 4.0.2, Scion, Frederick, MD, USA). The median gray value of the pixels from the entire photograph was automatically assigned by ImageJ software. These data were corrected by the tissue background signal. The quantification was performed by an experimenter blind to the code.

#### In Situ Hybridization of Thyroid Hormone Receptors

For *in situ* hybridization, a second group of naïve rats (n = 3 for each H and E groups) were sacrificed at P125 (50 days after surgery) and fixed by transcardial perfusion with 4% paraformaldehyde in 0.1 M PB. The brains were cryoprotected, frozen in dry ice, and serially sectioned on a cryostat at 25 µm in the coronal plane. The detection of TRβ1 and TRα1 mRNAs with ^35^S-labeled riboprobes at 1.6×10^7^ cpm/ml was performed in free-floating sections according to protocols previously described in detail [75]. Specific TRβ1 and TRα1 probes were designed based on the published rat cDNA sequences (accession no. J03819 and M18028, respectively). The following primers were used to amplify the TRβ1 specific sequence: forward 5′-CAGAAAAATGCCTTCCAGCCTG-3′ and reverse 5′-TCTCTTCGGTCTGGAAAGTCTG-3′. For TRα1 the primers were as follows: forward 5′-TCCACATGAAAGTCGAGTGCC-3′ and reverse 5′-CACACGGCCTTTCATAGCAAG-3′. The sections were mounted on coated slides, dehydrated, air dried, and exposed to Biomax MR film (Eastman Kodak, Rochester, NY, USA) for 17 days for TRβ1 and 11 days for TRα1 expression analysis. Autoradiographic films were scanned with a Coolscan II slide scanner (Nikon Corp., Tokyo, Japan) at a resolution of 1350 pixels/inch. The histological sections were Nissl stained and low magnification microphotographs (2×) were carried out with an Eclipse E400 microscope and Dn100 digital camera (both, Nikon Corp., Tokyo, Japan). The LA/BLA area identified in Nissl stained sections was drawn and superposed to the autoradiographic image. Densitometric quantification of mean grey values at the LA/BLA was performed following procedures peviously described [74], [76], using ImageJ software on three sections from every animal in both experimental groups. Similar measurements were made in the white matter to correct the data for tissue background hybridization signal.

### Statistical Analysis

All results were expressed as mean ± standard error of the mean (SEM) and significance of results was accepted at *p*≤0.05. The time each rat spent freezing was transformed to a percentage of freezing per minute. Statistical comparisons between groups were performed using Student's t-tests. To assess the statistical differences across sessions, repeated-measures generalized estimated equations were performed when appropriate (fixed effects: experimental group, session (repeated), group×session). The AUC of plasma corticosterone levels was calculated using the trapezoid rule. All analyses were performed using Statistics 18.0 system software (SPSS Inc., Chicago, IL, USA).
